# Genome- and peak-informed two-stage framework for scATAC-seq cell type identification

**DOI:** 10.1093/bioinformatics/btaf682

**Published:** 2025-12-27

**Authors:** Yan Liu, Sheng Guan, He Yan, Long-Chen Shen, Yiheng Zhu, Ji-Peng Qiang, Guo Wei

**Affiliations:** School of Information and Artificial Intelligence, Yangzhou University, Yangzhou, Jiangsu 225100, China; School of Information and Artificial Intelligence, Yangzhou University, Yangzhou, Jiangsu 225100, China; College of Information Science and Technology & Artificial Intelligence, Nanjing Forestry University, Nanjing, Jiangsu 210037, China; School of Computer Science and Engineering, Nanjing University of Science and Technology, Nanjing, Jiangsu 210094, China; College of Artificial Intelligence, Nanjing Agricultural University, Nanjing, Jiangsu 211800, China; School of Information and Artificial Intelligence, Yangzhou University, Yangzhou, Jiangsu 225100, China; School of Computer Science and Information Engineering, Bengbu University, Bengbu, Anhui 233030, China

## Abstract

**Motivation:**

Accurate cell type annotation is essential in scATAC-seq analysis, as it underpins the characterization of cellular heterogeneity, the identification of regulatory elements, and downstream biological discovery. However, current annotation methods still face major challenges. First, although some approaches attempt to integrate genomic sequence information, they typically rely on shallow sequence representations and thus fail to capture the long-range dependencies and regulatory signals encoded in DNA. Second, substantial batch effects introduced by different platforms, sequencing batches, or tissue sources remain insufficiently addressed. Existing models often lack robust distribution alignment and domain generalization capabilities, leading to confounding non-biological variation and reduced annotation accuracy across datasets.

**Results:**

To overcome these limitations, we propose *seqAlignATAC*, a two-stage intra-modality annotation framework that integrates sequence-derived embeddings with domain adaptation. In the first stage, we employ a large-scale pretrained nucleotide language model to extract low-dimensional, biologically informative representations from the genomic sequences of chromatin-accessible peaks. In the second stage, these embeddings are fed into a supervised neural network equipped with an adaptive alignment module to mitigate batch effects and harmonize feature distributions between labeled reference and unlabeled target datasets. Extensive experiments across multiple settings demonstrate that seqAlignATAC achieves competitive accuracy and robustness, effectively leveraging genome-level information while alleviating batch-induced distributional discrepancies.

**Availability and implementation:**

The source code of seqAlignATAC is available at: https://github.com/BioCS-Lab/seqAlignATAC.

## 1 Introduction

Single-cell Assay for Transposase-Accessible Chromatin using sequencing (scATAC-seq) is a high-throughput epigenomic technology that enables genome-wide profiling of chromatin accessibility at single-cell resolution (Rachid Zaim *et al.* 2024). By capturing cell-to-cell variability in accessible regulatory regions, scATAC-seq provides critical insights into the heterogeneity of gene regulatory potential—an essential dimension for elucidating cellular state transitions, developmental trajectories, and disease pathogenesis (Jansen *et al.* 2019, Lu *et al.* 2024). Among the key analytical challenges in scATAC-seq data interpretation, accurate cell type annotation stands out as a foundational step (Chen *et al.* 2019). It not only facilitates the deconvolution of complex cellular populations and their functional heterogeneity but also underpins the discovery of cell type-specific regulatory elements, transcription factor activity, and the functional consequences of non-coding genomic variation (Ma *et al.* 2023). As such, accurate cell type annotation bridges the gap between epigenomic profiling and functional genomics, enabling a deeper understanding of gene regulation in health and disease. Current scATAC-seq cell type annotation methods can be broadly divided into two categories based on the type of reference used. The first category employs annotated scRNA-seq datasets and transfers labels to scATAC-seq data via cross-modality mapping or joint embedding (Lin *et al.* 2022, Yan *et al.* 2023, Zhang *et al.* 2022b, Li *et al.* 2023). These methods build cell-level correspondence using shared features—such as gene activity scores (Granja *et al.* 2021)—or by projecting both modalities into a common latent space, as in scNCL (Yan *et al.* 2023), scCorrect (Liu *et al.* 2025), and scJoint (Lin *et al.* 2022). However, because scRNA-seq captures transcriptional output while scATAC-seq measures chromatin accessibility, the two modalities differ substantially in biological content, sparsity, and noise characteristics. Such discrepancies often lead to reduced accuracy and limited generalizability in cross-modality annotation. The second category instead relies on annotated scATAC-seq datasets and performs cell type prediction within the same modality (Berest and Tangherloni 2022). These intra-modality approaches—typically based on graph propagation, similarity inference, or transfer learning (Zhuang *et al.* 2021)—operate in a consistent epigenomic feature space and are generally more robust to platform variation. In this study, we follow the intra-modality paradigm and leverage high-quality annotated scATAC-seq references to achieve accurate and reliable cell type classification within the same modality.

Representative methods following the intra-modality annotation strategy include Cellcano (Ma *et al.* 2023), EpiAnno (Chen *et al.* 2022), scATAnno (Jiang *et al.* 2023), and SANGO (Zeng *et al.* 2024). Cellcano employs a two-stage supervised learning framework: initially, a multilayer perceptron (MLP) is trained on labeled reference data to predict cell types in the query dataset; subsequently, a self-distillation mechanism is applied to high-confidence “anchor” cells to refine predictions through pseudo-labeling. EpiAnno selects high-frequency chromatin accessibility peaks as input features and utilizes a nonlinear Bayesian neural network (Kononenko 1989) to model the latent cell distribution, incorporating uncertainty through probabilistic inference. scATAnno addresses the open-set annotation problem by introducing an uncertainty-aware scoring mechanism to detect cell types not present in the reference dataset, thereby improving generalization. SANGO extracts low-dimensional representations from peak-associated DNA sequences using a channel attention–based convolutional neural network (CA-CNN; Karthik *et al.* 2022), and integrates these representations into a graph transformer that propagates information across phenotypically similar cells to enhance annotation accuracy.

**Figure 1.**
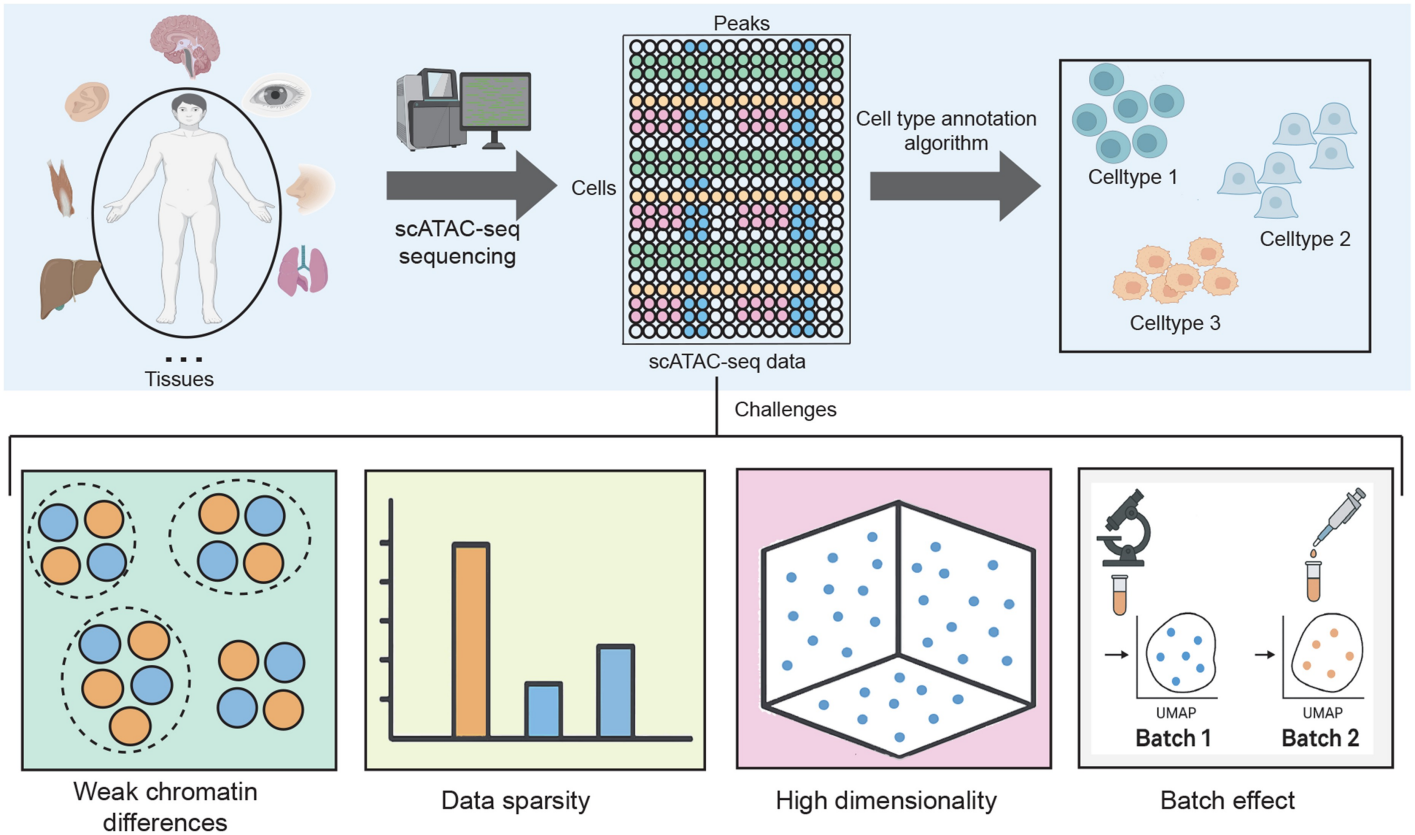
Illustration summarizing the overall workflow and challenges of scATAC-seq cell type annotation, including sparsity, batch effects, and weak chromatin differences.

Despite recent progress, scATAC-seq annotation remains difficult due to extreme sparsity, high noise, indirect regulatory signals, high dimensionality, and strong batch effects (Baek and Lee 2020) (Fig. 1). These issues hinder the extraction of reliable epigenetic patterns and weaken cross-dataset generalization. Sparse and noisy accessibility profile’s obscure meaningful biological structure, while batch effects introduce systematic biases across platforms. Thus, a major challenge is to obtain biologically informative representations from noisy data while simultaneously reducing cross-batch variation.

To address these limitations, we propose a two-stage framework for robust scATAC-seq cell type annotation. In stage one, a large-scale DNA sequence pretrained language model generates compact, informative embeddings from peak-associated genomic sequences, capturing both local and contextual regulatory signals. In stage two, these embeddings are processed by a supervised domain-adaptive network that aligns reference and query distributions while accounting for batch-specific biases. This integration of sequence-informed representation learning with adaptive alignment yields substantial gains in annotation accuracy and generalization.

In summary, this study contributes three key innovations. First, it directly leverages raw peak sequences and a pretrained model to obtain biologically meaningful embeddings, improving feature extraction under extreme sparsity. Second, it incorporates a supervised framework with adaptive alignment to mitigate batch effects across heterogeneous datasets. Third, it operates entirely within the scATAC-seq modality, avoiding the information loss and uncertainty inherent to cross-modality label transfer, thereby improving robustness and consistency of annotation.

## 2 Materials and methods

**Figure 2.**
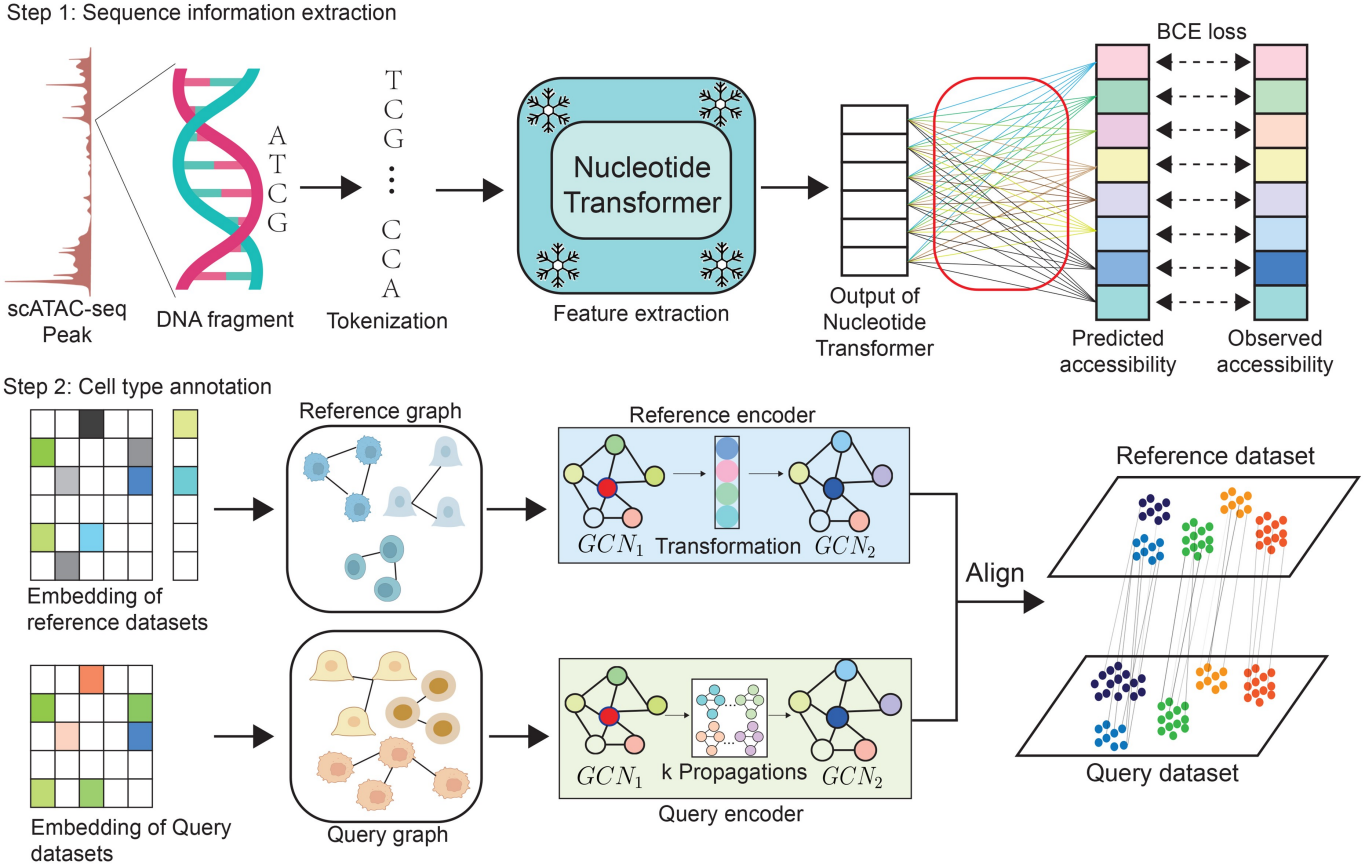
Overview of the proposed seqAlignATAC framework for cell type annotation from scATAC-seq data. (Step 1) *Feature extraction from genomic sequences*: Chromatin-accessible regions are tokenized and encoded using a pretrained DNA language model (DNA-LLM), generating contextualized embeddings that are used to predict accessibility profiles and derive cell embeddings. (Step 2) *Cell type annotation via graph domain adaptation*: Source and target cell embeddings are used to construct corresponding graphs.

To accurately model cellular heterogeneity and enable effective knowledge transfer across domains, this study proposes a two-stage framework for cell representation and classification (Fig. 2). In the first stage, genome sequence information and chromatin accessibility signals (peaks) are integrated, where high-dimensional gene embeddings are extracted using the Nucleotide TransformerNT (Dalla-Torre *et al.* 2025), and cell-level expression representations are aggregated from peak-level features. In the second stage, graphs are constructed for both the source and target domains based on the learned cell embeddings. A graph neural network (GNN) equipped with a graph domain adaptation (GDA) mechanism is then applied to perform cross-domain cell type prediction under unsupervised conditions. This method effectively combines sequence-level and structure-level representations while mitigating batch effects introduced by platform discrepancies, demonstrating strong cross-platform generalization performance.

### 2.1 Sequence information extraction

We employ the NT to perform high-fidelity representation learning on genomic sequences. Unlike traditional genome embedding techniques, such as k-mer frequency statistics, one-hot encoding, or shallow neural architectures, the NT is a large-scale, Transformer-based language model specifically tailored for DNA and RNA sequences. Trained in an unsupervised manner on extensive genomic corpora, it captures rich contextual semantics and long-range dependencies, enabling it to learn both local nucleotide patterns and global regulatory features, such as transcription factor binding sites and conserved sequence motifs. This results in significantly enhanced expressiveness and generalizability in sequence-level representations.

Moreover, the NT has demonstrated robust cross-species transferability and consistently outperforms conventional models in a range of genomic tasks, including gene function prediction, regulatory region identification, and variant effect annotation (Dalla-Torre *et al.* 2025). Within our framework, the model is used with fixed parameters and serves as a high-capacity feature extractor for chromatin accessibility peaks. The resulting sequence embeddings are integrated with experimentally observed ATAC-seq signals, which mark functionally active, open chromatin regions. This integration enables a more comprehensive and biologically informed modeling of the cellular regulatory landscape, ultimately supporting improved accuracy in downstream cell type classification. Specifically, we designed a feedforward neural network with *L* hidden layers to learn the mapping from genome embeddings to cell-level representations. The structure of the network is defined as follows:


(1) $$H^{\left(0\right)}=X$$

(2) $$\;H^{\left(l\right)}=\phi \left({\text{BN}}^{\left(l\right)}\left(H^{\left(l-1\right)}W^{\left(l\right)}+b^{\left(l\right)}\right)\right),l=1,2,\ldots ,L$$

(3) $$\hat{Y}=H^{\left(L\right)}W^{\left(L+1\right)}+b^{\left(L+1\right)}$$

where $X\in R^{N\times d}$ denotes the genome embedding input matrix, *N* is the number of genome sequences, and *d* is the embedding dimension for each genmoe. $W^{\left(l\right)}$ and $b^{\left(l\right)}$ represent the weight matrix and bias term of the *l*-th layer, respectively. ${\text{BN}}^{\left(l\right)}(\cdot )$ is the batch normalization operation for layer *l*, and ϕ(·) denotes the non-linear activation function. $\hat{Y}$ represents the model’s predicted peak value.

During model training, we use the Binary Cross Entropy as the optimization objective, defined as:


(4) $$L=-\frac{1}{N}\sum_{i=1}^{N}\left[y_{i}\;\text{log}\;\sigma ({\hat{y}}_{i})+(1-y_{i})\;\text{log}\;\left(1-\sigma ({\hat{y}}_{i})\right)\right]$$

where ${\hat{y}}_{i}$ is the predicted value for the *i*-th sample and $y_{i}$ is the corresponding ground-truth peak value.

After model training is completed, we extract the weight matrix $W^{\left(L+1\right)}$ from the output layer and use it as the embedding representation for each cell.

It is important to clarify why the output-layer weights correspond to cell-level embeddings rather than merely being learnable parameters. In our framework, each column $w_{c}$ of $W^{\left(L+1\right)}\in R^{D\times C}$ serves as the projection vector that maps peak-level latent representations $H^{\left(L\right)}$ into the predicted chromatin accessibility profile of cell *c*. Formally, the predicted accessibility of cell *c* is obtained as:


(5) $${\hat{A}}_{:,c}=H^{\left(L\right)}w_{c}$$

meaning that $w_{c}$ captures how cell *c* responds to different peak-level sequence features. During training, $w_{c}$ is optimized to best reconstruct the experimentally observed accessibility pattern of cell *c*, thereby encoding the regulatory preferences and accessibility determinants specific to that cell.

Thus, $W^{\left(L+1\right)}$ is not simply an output-layer parameter matrix; instead, its columns represent cell-specific regulatory signatures in the latent sequence-feature space. Each vector $w_{c}$ functions as a low-dimensional embedding that summarizes the unique chromatin accessibility landscape of cell *c*, providing a biologically meaningful and compact representation of cell identity.

### 2.2 Cell type annotation

As single-cell data are often generated across diverse platforms and research institutions, they are subject to substantial batch effects, which can significantly compromise the accuracy of downstream analyses. To address this challenge, we model the source and target datasets as two separate cell-cell similarity graphs, capturing the intrinsic structural relationships within each domain. Building upon these graph-based representations, we propose a domain-adaptive learning framework tailored for cell type classification. This framework is designed to effectively alleviate distributional discrepancies between the source and target domains, thereby enhancing the accuracy and robustness of cell type prediction in the target domain.

#### 2.2.1 Construction of graphs

After obtaining the cell embeddings, we adopt the *k*-Nearest Neighbors (kNN) algorithm to construct source and target domain graphs based on the similarity relationships in the embedding space. Specifically, the source domain graph is denoted as:


(6) $$G_{s}=(V_{s},E_{s},X_{s})$$

where $V_{s}$ represents the set of labeled source domain cells, $E_{s}$ denotes the edge set generated by computing distances between cells using their embeddings and constructing connections via the kNN algorithm (Cover and Hart 1967), and $X_{s}$ is the feature matrix of cells in the source domain.

Similarly, the target domain graph is defined as:


(7) $$G_{t}=(V_{t},E_{t},X_{t})$$

where $V_{t}$ is the set of unlabeled target domain cells, $E_{t}$ is constructed in the same way using cell embeddings and kNN, and $X_{t}$ is the feature matrix of target domain nodes.

#### 2.2.2 Adaptive cell classification module

To enable knowledge transfer from a labeled source graph to an unlabeled target graph, inspired by the previous works (Hamilton *et al.* 2017, Liu *et al.* 2024, Zhang *et al.* 2022a):

- The source and target graphs do not need to share the same GNN architecture;
- Stacking more propagation layers and reducing transformation layers on the target graph improves transfer performance.

Based on these insights, we design our model seqAlignATAC, which consists of three GCN modules and a domain-specific fully connected classifier head. This architecture enables robust feature abstraction and effective domain transfer. The core operation can be formulated as:


(8) $$\hat{Y}=f_{\text{cls}}(f_{\text{enc}}(X,G);\theta )$$

where $X\in R^{N\times d}$ is the input feature matrix, G=(V,E) is the input graph structure, and θ denotes shared model parameters between source and target graphs. The function $f_{\text{enc}}(\cdot )$ represents the graph encoder, and $f_{\text{cls}}(\cdot )$ denotes the classifier module.

In the source graph, due to full label supervision and the fact that cell data comes from the same platform and biological context, we adopt a shallow propagation strategy to avoid over-smoothing. The forward process is defined as:


(9) $$H_{s}=\text{ReLU}({\text{GCN}}_{1}(X_{s}))$$

(10) $$Z_{s}=\text{ReLU}(\text{Linear}(H_{s}))$$

(11) $${\hat{Y}}_{s}={\text{GCN}}_{3}(Z_{s})$$

where ${\text{GCN}}_{1}$ and ${\text{GCN}}_{3}$ are graph convolutional layers. The intermediate Linear operation is a depth-zero transformation (i.e. no propagation).

This shallow propagation design is due to the source domain having complete label supervision, where node semantic information is already clear and reliable. Using too many propagation layers in the source domain could lead to over-smoothing, weakening class separability, and amplifying noise, especially in cases of erroneous edges.

In contrast, for the target graph, which lacks labels, we adopt a deep propagation strategy to fully exploit the contextual information. We stack *K* propagation layers; the corresponding computation is defined as:


(12) $${\hat{Y}}_{t}={\text{GCN}}^{\left(K\right)}(X_{t})$$

In the target graph, we adopt a deep propagation strategy to fully extract contextual information from the graph structure. The propagation process is defined as:


(13) $$H_{t}=\text{ReLU}({\text{GCN}}_{1}(X_{t}))$$

(14) $$Z_{t}={\text{GCN}}_{2}^{\left(K\right)}(H_{t})$$

(15) $${\hat{Y}}_{t}={\text{GCN}}_{3}(Z_{t})$$

Here, ${\text{GCN}}_{2}^{\left(K\right)}$ denotes *K* successive propagation operations performed in the second graph convolution layer, effectively enlarging the receptive field and enhancing the feature expressiveness of nodes in the target graph.

The deep propagation in the target domain is designed to leverage broader neighborhood information to establish semantic consistency in the absence of labels, enhancing the alignment across domains. Additionally, deep propagation improves feature smoothness and robustness, mitigating the challenges posed by noisy or sparse data in the target domain.

During the training process, the source and target domains share all GCN layer weights and classifier parameters to ensure feature alignment and consistency between domains. This architectural design directly implements the two core strategies: enabling flexible domain adaptation through asymmetric design, while enhancing message propagation in the target graph to compensate for the lack of supervision. Together, these mechanisms contribute to superior performance in unsupervised GDA.

#### 2.2.3 Loss function

To realize knowledge transfer from the labeled source domain to the unlabeled target domain, we jointly optimize the classification loss and domain adversarial loss during training. The overall loss is defined as follows:

We perform cell classification on the labeled source graph $G_{s}=(V_{s},E_{s})$ using Negative Log-Likelihood Loss (NLLLoss). Given the predicted label probability ${\hat{Y}}_{s}$ and ground truth $Y_{s}$, the classification loss is defined as:


(16) $$L_{\text{cls}}=\text{NLLLoss}(\text{log}\;\text{Softmax}({\hat{Y}}_{s}),Y_{s})$$

where ${\hat{Y}}_{s}=f_{\text{cls}}(f_{\text{enc}}(X_{s},G_{s});\theta )$ denotes the output from the classification module on the source graph.

To minimize the feature distribution gap between source and target domains, we adopt a domain adversarial strategy that incorporates a gradient reversal layer (GRL) (Osumi *et al.* 2019) and a domain discriminator (Liu *et al.* 2024).

Let $Z_{s}=f_{\text{enc}}(X_{s},G_{s})$ and $Z_{t}=f_{\text{enc}}(X_{t},G_{t})$ be the encoded features from source and target domains, respectively. After applying GRL, we obtain:


(17) $$D_{s}=f_{\text{dom}}(\text{GRL}(Z_{s})),\;D_{t}=f_{\text{dom}}(\text{GRL}(Z_{t}))$$

We concatenate the outputs and construct domain labels:


(18) $$D=[D_{s};D_{t}],\;Y_{d}=[\underset{N_{s}}{\underset{︸}{0,\ldots ,0}},\underset{N_{t}}{\underset{︸}{1,\ldots ,1}}]$$

The domain adversarial loss is then defined by the cross-entropy function:


(19) $$L_{\text{dom}}=\text{CrossEntropy}(D,Y_{d})$$

We dynamically adjust the adversarial weight γ based on training progress:


(20) $$\gamma =\frac{2}{1+\text{exp}(-10\cdot p)}-1,\;p=\frac{e}{E}$$

where *e* is the current training epoch and *E* is the total number of epochs. The total loss is a weighted sum of the classification and domain adversarial losses:


(21) $$L_{\text{total}}=L_{\text{cls}}+\lambda \cdot L_{\text{dom}}$$

where λ is a hyperparameter that balances the domain adversarial loss.

## 3 Results

### 3.1 Datasets and baseline methods

We provide detailed descriptions of the datasets used in this study, the data preprocessing procedures, and the parameter settings of all baseline methods in the Supplementary Materials, available as supplementary data at *Bioinformatics* online. Please refer to the Datasets, Baseline Methods, and Data Preprocessing sections of the Supplementary Materials, available as supplementary data at *Bioinformatics* online for full details.

### 3.2 Cross-platform cell type annotation

We evaluated the performance of various cell type annotators in the Intra-Platform Cell Type Annotation section of the Supplementary Materials, available as supplementary data at *Bioinformatics* online, highlighting the performance of seqAlignATAC in intra-platform scenarios. To evaluate the robustness of cell type annotation across sequencing platforms, we performed bi-directional transfer experiments between snATAC-seq and sciATAC-seq datasets, as summarized in Tables 1 and 2. Table 1 reports cross-platform results between snATAC-seq datasets (MosA1, MosM1, MosP1) and sciATAC-seq datasets (WholeBrainA, WholeBrainB), while Table 2 extends the analysis by incorporating the 10x Genomics-based *MouseBrain(10x)* dataset as either the reference or the query.

**Table 1.** Cross-platform cell type annotation between snATAC-seq and sciATAC-seq.

Method	R: MosA1 Q: WholeBrainA		R: WholeBrainA Q: MosA1		R: MosP1 Q: WholeBrainA		R: WholeBrainA Q: MosP1		R: MosM1 Q: WholeBrainA		R: WholeBrainA Q: MosM1	
	acc	F1	acc	F1	acc	F1	acc	F1	acc	F1	acc	F1
SANGO	0.651	**0.587**	0.696	0.298	0.707	0.608	0.606	0.294	0.587	0.539	0.645	0.295
scNym	0.682	0.584	0.747	0.311	0.694	0.599	0.664	0.293	0.680	0.599	0.690	0.296
scJoint	0.645	0.568	0.757	0.340	0.557	0.524	0.708	0.349	0.664	0.592	0.748	0.355
Cellcano	0.642	0.539	0.534	0.304	0.641	0.535	0.469	0.295	0.641	0.529	0.451	0.293
seqAlignATAC	**0.701**	0.578	**0.824**	**0.582**	**0.713**	**0.618**	**0.729**	**0.373**	**0.803**	**0.697**	**0.759**	**0.380**

Bold values now represent the best performance within each group.

**Table 2.** Cross-platform cell type annotation between MouseBrain (10×) and sciATAC-seq datasets.

Method	R: MouseBrain (10×) Q: WholeBrainA		R: WholeBrainA Q: MouseBrain (10×)		R: MouseBrain (10×) Q: WholeBrainB		R: WholeBrainB Q: MouseBrain (10×)	
	acc	F1	acc	F1	acc	F1	acc	F1
SANGO	0.731	0.631	0.702	0.361	**0.701**	**0.627**	0.700	0.360
scNym	0.664	0.547	**0.757**	0.366	0.663	0.575	**0.779**	0.371
scJoint	0.710	0.615	0.745	0.354	0.681	0.613	0.761	0.358
Cellcano	0.632	0.525	0.698	0.371	0.605	0.509	0.680	0.364
**seqAlignATAC**	**0.869**	**0.710**	0.742	**0.486**	0.694	0.618	0.693	**0.377**

Bold values now represent the best performance within each group.

Across all transfer directions, our method seqAlignATAC consistently outperforms baseline approaches in both accuracy and macro-F1. For example, in the challenging *WholeBrainA* →  *MosA1* task (Table 1), it achieves 0.824 accuracy and 0.582 macro-F1, significantly surpassing scJoint (0.757/0.340) and scNym (0.747/0.311).

Similarly, on the *MouseBrain(10x)* dataset (Table 2), seqAlignATAC attains the best performance, with a macro-F1 of 0.377 in the *WholeBrainB* →  *MouseBrain(10x)* task, highlighting its robustness across diverse sequencing platforms and cell profiles.

These findings underscore two key insights:

1. Peak-based methods (e.g. scNym, scJoint, Cellcano) demonstrate moderate performance but exhibit limited generalization across platforms, particularly in reverse transfer settings.
2. Sequence-aware models (e.g. SANGO) leverage DNA-level information for improved robustness but still fall short of seqAlignATAC. In contrast, seqAlignATAC effectively integrates sequence-derived representations with domain adaptation, yielding substantial and consistent performance gains.

In conclusion, seqAlignATAC exhibits strong cross-platform transferability, particularly in scenarios involving heterogeneous sequencing protocols such as snATAC-seq and sciATAC-seq. These results validate the utility of combining local chromatin accessibility signals with global DNA sequence context for robust and accurate cell type annotation.

### 3.3 Contribution of the sequence feature extraction module

To evaluate the effectiveness of the sequence feature extraction module (Nucleotide Transformer embeddings), we performed an ablation experiment in which all sequence-derived information was removed. Specifically, instead of using peak-level sequence embeddings, we constructed cell representations directly from the cell-by-peak matrix. The matrix was first binarized to indicate peak presence or absence, followed by TF-IDF (Aizawa 2003) normalization to balance peak occurrence across cells. Finally, we applied Truncated SVD (Hansen 1987) to obtain a 128-dimensional latent space (LSI), which was then fed into the second-stage graph domain adaptation module for prediction.

**Figure 3.**
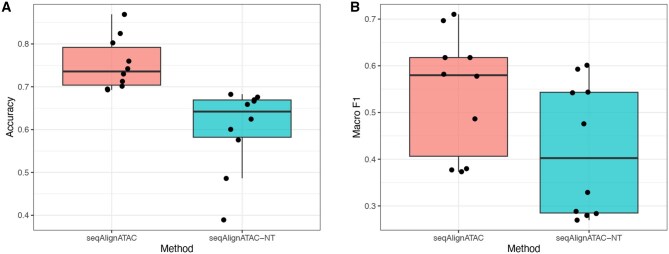
Ablation study of the sequence feature extraction module. (A) Accuracy and (B) macro F1 of the full model (seqAlignATAC) versus the variant without sequence embeddings (seqAlignATAC–NT). Removing sequence-derived features leads to a notable decrease in performance, indicating the critical contribution of Nucleotide Transformer embeddings to cell-type annotation accuracy.

As shown in Fig. 3, removing sequence features (seqAlignATAC–NT) leads to a clear performance drop across multiple dataset pairs. Both accuracy and macro F1 are substantially lower compared with the full model (seqAlignATAC), demonstrating that peak-level sequence embeddings provide essential biological information and significantly contribute to the overall performance of our framework.

## 4 Computational efficiency and scalability

In addition to the main experimental results, we conducted a comprehensive analysis of the computational efficiency, scalability. Specifically, we report detailed measurements of runtime and memory consumption across the three major stages of the framework, compare end-to-end efficiency with widely used baseline methods, and evaluate performance on a large-scale cross-tissue dataset comprising over 120,000 cells. All results, tables, and methodological details are provided in the section Computational Efficiency and Scalability Analysis of Supplementary Materials, available as supplementary data at *Bioinformatics* online.

## 5 Ablation experiments

To systematically validate the design choices of seqAlignATAC, we conducted a comprehensive set of ablation studies covering multiple aspects of the framework, including the sensitivity of the *k*-nearest neighbor construction, the contribution of the sequence feature extraction module, the impact of the domain adaptation mechanism and asymmetric propagation depth, robustness under perturbed DNA sequences, and comparisons with mainstream domain alignment and GRL scheduling strategies. Detailed experimental settings, results, and figures for all ablation analyses are provided in the Ablation experiments section of Supplementary Materials, available as supplementary data at *Bioinformatics* online.

## 6 Discussion

Our study presents seqAlignATAC, a domain-adaptive and sequence-aware framework for accurate cell type annotation from scATAC-seq data. Extensive experiments across diverse intra-modality tasks show that seqAlignATAC outperforms existing methods, owing to two main innovations: (i) biologically meaningful embeddings from a pretrained DNA language model, and (ii) a GDA module that mitigates distribution shifts.

Ablation studies confirm the critical role of domain adaptation. Comparing *Embedding(NT)+DA* with *Embedding(NT)+KNN*, the DA component consistently boosts accuracy and macro-F1, especially in challenging cross-tissue or cross-platform settings. For instance, on *MosA1* →  *WholeBrainB*, DA yields 0.9015 accuracy and 0.7694 macro-F1, greatly surpassing KNN. Gains are particularly notable for rare cell types, highlighting the DA module’s ability to amplify weak biological signals and reduce batch effects.

Looking ahead, there are several promising directions to further enhance seqAlignATAC. First, adapting the framework for cross-modality alignment (e.g. aligning scATAC-seq to scRNA-seq) would significantly expand its versatility. Second, incorporating biological priors, such as transcription factor binding motifs or gene regulatory networks, could improve both the interpretability and biological relevance of the model’s predictions. Third, as DNA language models continue to evolve, task-specific fine-tuning could further optimize the quality of sequence embeddings. Finally, uncertainty estimation could be integrated to increase the robustness of the model, particularly when dealing with noisy data or previously unseen cell populations.

## Supplementary Material

btaf682_Supplementary_Data

## Data Availability

A data availability statement has been included in the manuscript. The data supporting the findings of this study are publicly available in the Synapse repository at https://www.synapse.org/Synapse:syn52559388/files/.
