# Supplementary material for: Genome- and peak-informed two-stage framework for scATAC-seq cell type identification
Source: Bioinformatics. 2025 Dec 27;42(2):btaf682. doi: 10.1093/bioinformatics/btaf682 (PMC12930843; doi:10.1093/bioinformatics/btaf682)
Supplement: btaf682_Supplementary_Data [file btaf682_supplementary_data.pdf]

Datasets

To thoroughly validate the effectiveness of our proposed approach, we adopted a collection of publicly accessible single-nucleus ATAC-seq (snATAC-seq) datasets derived from various sequencing platforms and tissue regions. Among them, the MosA1, MosM1, and MosP1 datasets represent chromatin accessibility measurements from different functional subregions of the mouse cerebral cortex. These data are aligned to the GRCm38 mouse reference genome and can be obtained from the GEO repository under accession number `GSE126724`.

In addition, we included the Mouse Brain (10x) dataset, which was produced using the 10x Genomics technology and aligned to the `mm10` reference genome. This dataset is publicly available at: `https://www.10xgenomics.com/resources/adult_brain_fresh_5k`.

We further incorporated two large-scale sciATAC-seq datasets, WholeBrainA and WholeBrainB, which provide atlas-level chromatin accessibility profiles of the entire mouse brain. These datasets were annotated using the `mm9` genome build and are accessible either through GEO (accession: `GSE111586`) or via the following website: `http://atlas.gs.washington.edu/mouse-atac/data/`. We additionally incorporated two large non-brain tissue datasets—heart (79,248 cells) and intestine (42,942 cells), yielding a combined total of 122,190 cells. These datasets, sourced from the public accession GSE149683, exhibit biological characteristics that differ markedly from the brain-focused data used earlier. This allows us to further assess the robustness and generalizability of our method under distinct tissue contexts.

Baseline methods

To assess the performance of our method, we benchmark it against four established baseline approaches. Among them, scNymKimmel and Kelley (2021), scJointLin et al. (2022), and CellcanoMa et al. (2023) primarily leverage peak-level chromatin accessibility features, without explicitly utilizing genome sequence information. For scJoint, we adapt its use to the scATAC-seq setting, ensuring that both training and inference are conducted exclusively with scATAC-seq data. Cellcano is evaluated in accordance with its original implementation: when the number of target cells is limited, predictions from the first training stage are reported; otherwise, results from the second stage are adopted. On the other hand, SANGOZeng et al. (2024) incorporates both chromatin accessibility peaks and associated genome sequences to derive joint representations through a hybrid model. scATAnnoJiang et al. (2023) introduces an automated workflow for cell type annotation. In addition to perform cell type annotation, the method provides two uncertainty scoring mechanisms: a k-nearest-neighbors (KNN)Cover and Hart (1967)–based score and a weighted-distance–based score. In our study, we used the default parameter settings. For consistency and fairness, all datasets used in the baseline comparisons are preprocessed using the same pipeline employed by SANGO.

Data Preprocessing

In the cross-platform experiments, we first constructed a common peak set by merging the two datasets of ATAC-seq data from different sources. Specifically, we merged the two datasets and counted the frequency of each peak across cells. A threshold based on the proportion of peak occurrences (default is 1% of the total number of cells) was applied to filter the peak set. Peaks that met the threshold criteria were retained in both the source and target datasets to ensure consistency in the peak space during cross-platform analysis.

Regarding sequence feature extraction, we did not truncate or re-pad the sequences, but instead, we retained the original genomic sequence lengths corresponding to the ATAC-seq peaks and directly input them into the Nucleotide Transformer to generate the corresponding genome embeddings. We used the tokenization method built into Nucleotide Transformer to encode the DNA sequences, aiming to preserve the biological integrity of the sequences to the greatest extent. Additionally, in this study, we did not filter genomic blacklist regions such as those provided by ENCODE. This decision was made because the original peaks were generated through standard pipelines in each dataset, and since we used a common peak set, no additional blacklist removal step was necessary.

Details of Model Structure and Training Parameter Settings

In the cell representation learning stage, we employ a three-layer fully connected neural network with hidden layer dimensions of 512, 256, and 128, respectively. The learning rate is set to 0.001, and the model is trained for 350 epochs. In the adaptive cell classification module, the learning rate is uniformly set to 0.0025, and the hidden layer size of the graph convolutional network (GCN) is set to 200. The weight coefficient λ for the adversarial loss is fixed at 0.5.

No propagation layers are used in the source graph, whereas a 15-layer propagation structure is employed for the target graph to capture higher-order neighborhood information. The number of training epochs for the adaptive module varies based on specific training–testing dataset combinations as follows: 67 epochs when the training set is *MouseBrain (10x)* and the testing set is *WholeBrainA*, or the training set is *WholeBrainB* and the testing set is *MosA1*; 180 epochs when the training set is *MosA1* and the testing set is *WholeBrainB*, or the training set is *MosM1* and the testing set is *WholeBrainA*; and 8 epochs for all other dataset combinations. These configurations are empirically determined to balance convergence efficiency and generalization across diverse experimental conditions.

We used symmetric normalization for the graph convolutions, which is standard practice in GCN models. Specifically, the adjacency matrix is normalized as follows:

$$\hat{A} = D^{-\frac{1}{2}}(A + I)D^{-\frac{1}{2}}, \tag{1}$$

where D is the degree matrix and I is the identity matrix. This helps stabilize gradient propagation and reduces the imbalance caused by varying node degrees.

For the GCN architecture, we did not employ other variants like GCNIIChen et al. (2020), GraphSAGEHamilton et al. (2017), or GATVeličković et al. (2017). The main innovation of our framework lies in the "shallow propagation in the source domain and deep propagation in the target domain" mechanism, designed to handle the differences in the source and target domains. In the target domain, deep propagation helps capture more contextual information, while shallow propagation in the source domain prevents over-smoothing.

To prevent overfitting, we applied common regularization techniques such as dropout with a ratio of 0.1 and weight decay with a value of 0.001.

For handling class imbalance, we used SMOTE Fernández et al. (2018) oversampling on the source domain to increase the number of samples for underrepresented cell types, helping the model better recognize rare cell types.

Intra-Platform Cell Type Annotation

To assess the effectiveness of SeqAlignATAC in low-domain-shift scenarios, we performed intra-platform experiments where the reference and query datasets were derived from the same sequencing platform, thereby minimizing variability due to platform-specific biases. This experimental scenario is comparatively less challenging, as it minimizes the domain shift caused by variations in sequencing technology, batch effects, or library preparation protocols. Consequently, this setup serves as a baseline environment to evaluate the robustness and stability of annotation models when the input data distribution is relatively homogeneous.

As shown in Table 1, our proposed method, SeqAlignATAC, consistently achieves strong performance across all intra-platform tasks, outperforming or matching other state-of-the-art methods in terms of both accuracy and F1-score. In particular, SeqAlignATAC reaches near-perfect annotation results in tasks such as MosM1 → MosA1 (Accuracy: 0.980, F1: 0.979) and MosP1 → MosA1 (Accuracy: 0.979, F1: 0.978). Moreover, its performance remains highly stable across all transfer directions, with minimal degradation observed, underscoring its robust generalization ability under low domain discrepancy.

In contrast, baseline methods such as Cellcano and scNym exhibit greater variability across tasks. For instance, Cellcano shows notably lower performance in the WholeBrainB → WholeBrainA setting (Accuracy: 0.866, F1: 0.701), which suggests sensitivity to even subtle within-platform distributional shifts. While some methods like scJoint also demonstrate strong results, SeqAlignATAC shows a more consistent superiority in maintaining high performance across all tested scenarios.

Overall, these results demonstrate that SeqAlignATAC not only achieves state-of-the-art accuracy but also exhibits exceptional stability in intra-platform settings. This suggests that the method is inherently well-suited for clean and aligned datasets, and lays a strong foundation for tackling more complex cross-platform or cross-modality annotation tasks in subsequent experiments.

Table 1. Intra-Platform performance comparison of different methods.

Method	R: WholeBrainA Q: WholeBrainB		R: WholeBrainB Q: WholeBrainA		R: MosA1 Q: MosM1		R: MosM1 Q: MosA1		R: MosA1 Q: MosP1		R: MosP1 Q: MosA1		R: MosM1 Q: MosP1		R: MosP1 Q: MosM1	
	**Acc**	**F1**	**Acc**	**F1**	**Acc**	**F1**	**Acc**	**F1**	**Acc**	**F1**	**Acc**	**F1**	**Acc**	**F1**	**Acc**	**F1**
SANGO	0.936	0.790	0.915	**0.863**	**0.978**	**0.975**	0.976	0.975	**0.977**	**0.978**	0.977	**0.979**	**0.980**	**0.975**	0.963	0.962
scNym	0.912	0.815	0.897	0.778	0.952	0.943	0.964	0.956	0.955	0.952	0.964	0.958	0.959	0.946	0.958	0.945
scJoint	**0.942**	**0.841**	0.920	0.801	0.973	0.968	0.979	0.976	0.966	0.968	0.974	0.972	0.976	0.973	**0.973**	**0.966**
Cellcano	0.885	0.726	0.866	0.701	0.921	0.900	0.923	0.906	0.923	0.897	0.923	0.906	0.926	0.899	0.925	0.900
SeqAlignATAC	0.930	0.837	**0.923**	0.852	0.969	0.963	**0.980**	**0.979**	0.967	0.964	**0.979**	0.978	0.972	0.965	0.968	0.958

Computational Efficiency and Scalability Analysis

Computational Efficiency analysis

To better understand the computational characteristics of the proposed seqAlignATAC framework, we decomposed the entire pipeline into three major stages: (1) sequence encoding using the Nucleotide Transformer, (2) cell-level feature construction, and (3) graph construction followed by domain adaptation training. Table 2 summarizes the time and memory requirements of each stage on the MosM1 → WholeBrainA task.

The results indicate that the majority of the computational cost arises from the sequence encoding stage, which requires approximately 138 minutes and 3.2 GiB of GPU memory. This step involves forward inference through a large-scale DNA language model and is executed only once to produce high-quality peak-level embeddings. In contrast, the subsequent stages are significantly more efficient: cell embedding requires only 21 minutes, while graph-based domain adaptation takes less than 30 seconds, demonstrating that the computational bottleneck is heavily concentrated in the sequence feature extraction process rather than in the graph learning module.

To contextualize the efficiency of seqAlignATAC, we further compared its end-to-end runtime with several widely used baseline methods under the same hardware environment (Table 3). As expected, seqAlignATAC exhibits longer total training time than lightweight methods such as scJoint and scNym, primarily due to the additional cost of generating high-dimensional

Table 2. Computation time and memory usage of the three main stages of seqAlignATAC on the MosM1 → WholeBrainA task.

**Stage**	**Time**	**Memory**
Sequence encoding	138 min 17 s	3290 MiB
Cell embedding	21 min 15 s	698 MiB
Graph + Domain adaptation	24 s	736 MiB

Table 3. Comparison of total training time between seqAlignATAC and baseline methods under the same GPU environment (Cellcano runs on CPU).

**Method**	**MosM1 → WholeBrainA**	**MouseBrain(10x) → WholeBrainA**
sango	145 min 16 s	107 min 8 s
scNym	7 min 33 s	5 min 35 s
scJoint	3 min 11 s	3 min 14 s
Cellcano (CPU)	11 min 55 s	5 min 4 s
seqAlignATAC (ours)	159 min 42 s	152 min 14 s

Table 4. Performance comparison on large-scale cross-tissue datasets (122,190 cells).

**Method**	**Intestine → Heart**				**Heart → Intestine**			
	Acc	F1	Time	Memory	Acc	F1	Time	Memory
sango	0.4976	0.1666	223m25s	1656 MiB	0.6571	0.2217	223m37s	1656 MiB
scNym	0.6788	0.2924	26m31s	1094 MiB	0.6325	0.2908	44m10s	1094 MiB
scJoint	0.6577	0.2560	35m03s	2742 MiB	0.6958	0.2779	38m54s	2742 MiB
Cellcano (CPU)	0.2471	0.0701	26m10s	—	0.2371	0.1513	50m48s	—
**seqAlignATAC**	**0.7025**	**0.4009**	480m37s	3290 MiB	**0.7175**	**0.3001**	599m17s	3290 MiB

sequence embeddings. However, the performance gains obtained from integrating cross-modality peak sequence information and the robustness improvements in cross-platform alignment justify this additional computational expense.

Notably, despite the higher cost of sequence encoding, the graph-based domain adaptation stage of seqAlignATAC remains extremely fast (20–30 seconds), highlighting that the method is scalable for large datasets once sequence embeddings are precomputed. This design reflects a practical trade-off: investing computational resources upfront to obtain rich, biologically informed representations leads to superior cross-domain performance during downstream annotation tasks.

Large-Scale Dataset Evaluation

To address the reviewer's concern regarding the predominance of small-scale datasets in the original experiments, we additionally conducted extensive evaluations on two large non-brain tissues—heart (79,248 cells) and intestine (42,942 cells), resulting in a combined dataset of 122,190 cells. These data were obtained from the public dataset GSE149683 and differ substantially from the original brain-related datasets.

We performed two cross-tissue transfer settings: intestine → heart and heart → intestine. The results (Table 4) demonstrate that seqAlignATAC maintains strong performance and stability even at the scale of over one hundred thousand cells. In both directions, our method achieves the highest F1-score among all compared baselines, including scNym, scJoint, and Cellcano. Moreover, seqAlignATAC consistently shows superior generalization when transferring between distinct biological systems (digestive vs cardiac tissues), indicating robust cross-tissue adaptation capability.

Evaluation on Unknown Cell Type Detection

To further assess the ability of seqAlignATAC to identify previously unseen cell types, we performed experiments on two cross-platform datasets: MosA1 → WholeBrainA and MosP1 → WholeBrainA. In both settings, we manually removed the cell type *Inhibitory Neurons* from the source-domain training data (MosA1 or MosP1), and treated it as an unknown class during inference. This setup provides a realistic evaluation scenario in which the model must detect and label novel cell populations that do not appear in the annotated reference dataset.

For seqAlignATAC, we adopted a probability threshold of 0.65 to identify unknown cells. For scNym, we followed the same threshold (0.65). For scATAnno, we used its default hyperparameters for unknown prediction. Unknown-cell detection performance was quantified using three metrics: *precision of unknown prediction*, *recall (detection rate)*, and *F1-score*, defined as:

$$\text{Precision} = \frac{\text{Correctly predicted unknown cells}}{\text{Predicted unknown cells}} \tag{2}$$

$$\text{Recall} = \frac{\text{Correctly predicted unknown cells}}{\text{True unknown cells}} \tag{3}$$

$$\text{F1} = \frac{2 \cdot (\text{Precision} \cdot \text{Recall})}{\text{Precision} + \text{Recall}} \tag{4}$$

Table 5. Performance on unknown cell type detection after removing "Inhibitory Neurons" from the training data.

Method	MosA1 → WholeBrainA			MosP1 → WholeBrainA		
	Prec	Recall	F1	Prec	Recall	F1
scNym	0.8563	0.1704	0.2843	0.8283	0.0977	0.1748
seqAlignATAC	**0.6577**	**0.7902**	**0.7179**	**0.6346**	**0.7306**	**0.6792**
scATAnno	0.2205	0.3099	0.2577	0.2862	0.2193	0.2483

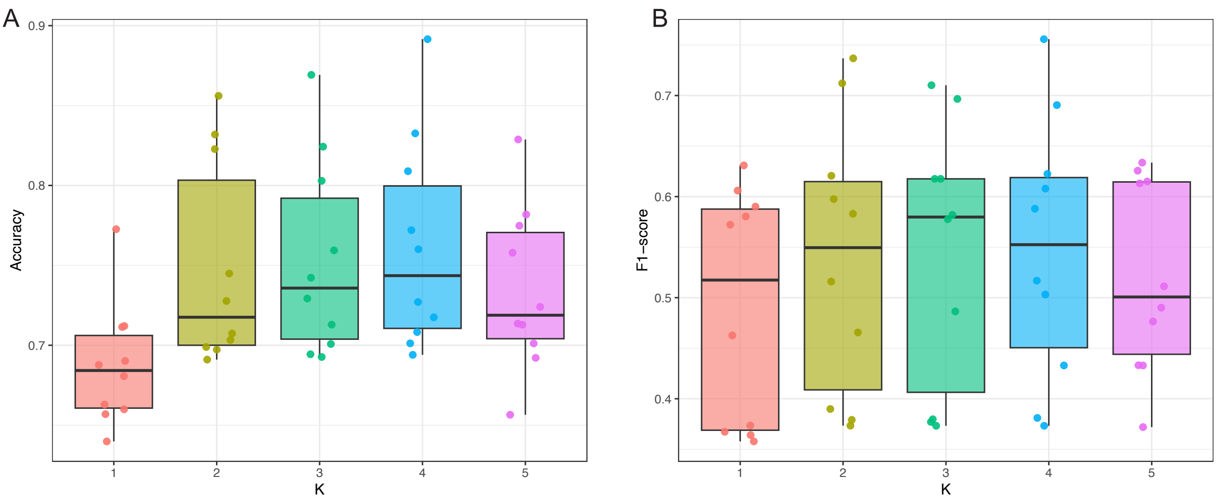

Fig. 1. Sensitivity analysis of the number of neighbors k in k-NN graph construction. (**A**) Accuracy and (**B**) macro F1 across multiple cross-domain cell annotation tasks, evaluated under different k values (1–5).

Using only precision can be misleading, for example, a model that predicts only one cell as unknown (and happens to be correct) would achieve 100% precision but fail to detect the full unknown population. Therefore, the F1-score provides a fairer and more comprehensive evaluation of unknown detection.

Table 5 summarizes the results. Detecting unseen cell types is inherently challenging for all evaluated methods. Nevertheless, seqAlignATAC achieves the **highest F1-score** on both datasets, demonstrating its superior capability to identify novel biological populations in cross-platform single-cell ATAC-seq data. This advantage likely arises from its integration of DNA-sequence-informed peak embeddings and graph-based domain adaptation, which jointly facilitate more discriminative boundary formation for unknown cell populations.

Ablation experiments

Number of Neighbors Sensitivity Analysis

To investigate the influence of the number of neighbors used in the k-nearest neighbor (k-NN) graph construction, we conducted a systematic sensitivity analysis by varying k from 1 to 5 (default $k = 3$). As shown in Figure 1, increasing k from 1 to 3 generally leads to consistent improvements in both accuracy and macro F1 across most cross-domain annotation tasks. This trend indicates that using a slightly larger neighborhood helps stabilize the graph structure and enhances local feature aggregation.

However, when $k > 3$, the performance on several dataset pairs shows minor fluctuations or slight decreases. We attribute this to the introduction of potentially noisy or biologically irrelevant edges when too many neighbors are included, which may weaken the domain-specific topological structure. Overall, the results support $k = 3$ as a balanced and robust choice for constructing cross-domain cell graphs.

Boundary Case Analysis under Low-Quality DNA Sequences

To evaluate the robustness of our method under low-quality or noisy DNA sequences, we conducted a boundary case analysis by introducing controlled perturbations to the input sequences during the sequence-embedding stage. Specifically, we randomly shuffled 10%, 20%, and 30% of the ATCG nucleotides to simulate sequencing errors, noise, or degraded sequence quality, while keeping all other components of the model unchanged.

As shown in Figure 2, increasing the degree of sequence perturbation consistently leads to decreases in both accuracy and macro F1 across multiple cross-platform tasks. In contrast, the unperturbed condition (*Correct*) achieves the highest performance, indicating that the model remains stable and effective when provided with normal-quality DNA sequences. These results demonstrate that although performance degrades under severe sequence corruption, seqAlignATAC retains reasonable robustness and benefits substantially from high-quality sequence information.

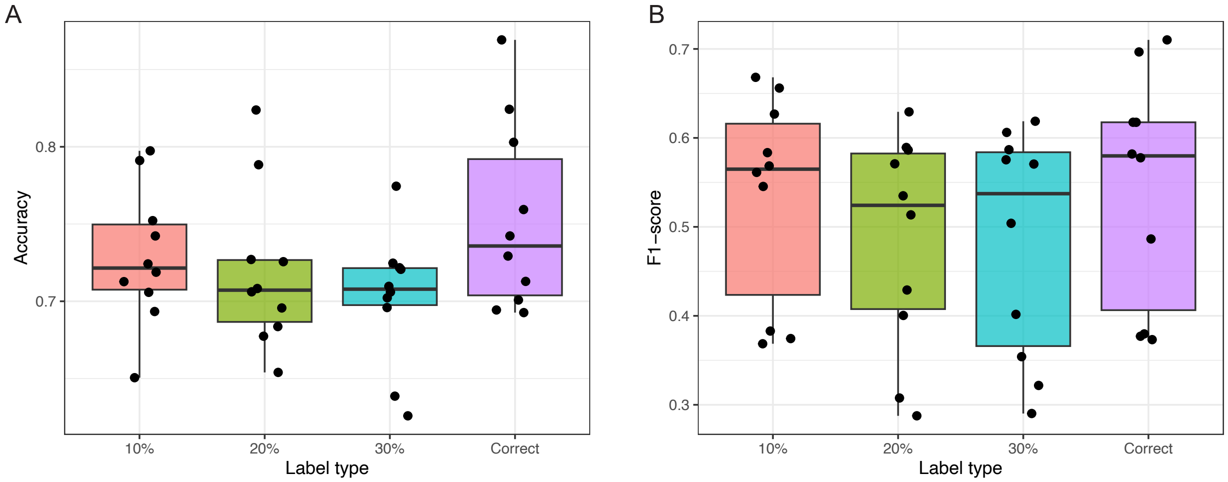

Fig. 2. Impact of DNA sequence perturbation on cross-domain annotation performance. (**A**) Accuracy and (**B**) macro F1 under different perturbation levels (10%, 20%, 30% shuffled nucleotides) compared with the unperturbed condition (*Correct*). Performance degrades as more nucleotides are shuffled, highlighting the importance of high-quality sequence information for robust representation learning.

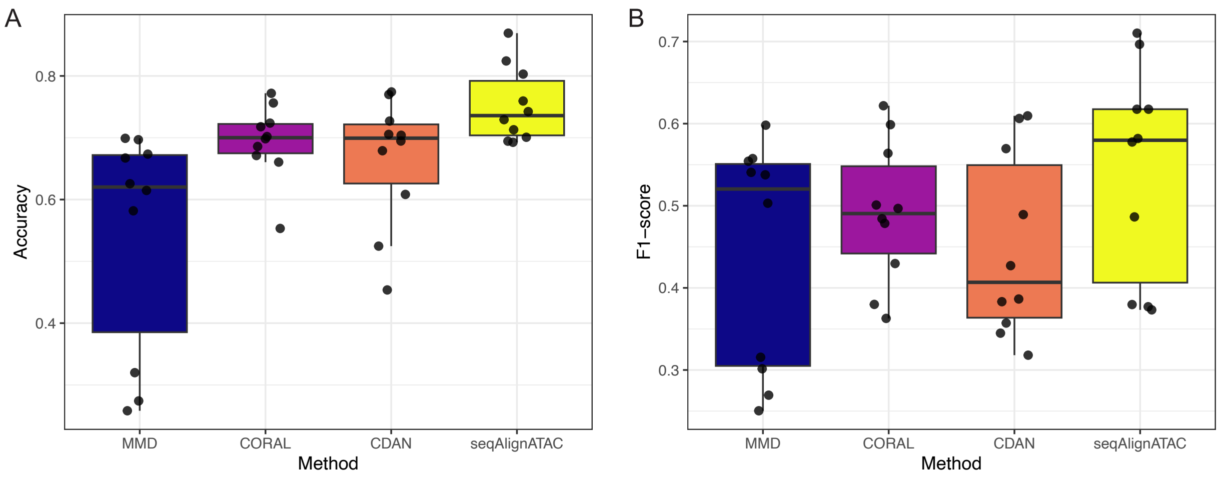

Fig. 3. Comparison of domain alignment strategies. (**A**) Accuracy and (**B**) macro F1 for MMD, CORAL, CDAN, and the proposed seqAlignATAC across multiple cross-platform annotation tasks.

Comparison with Mainstream Domain Alignment Strategies

To further evaluate the effectiveness of our domain alignment strategy, we compared the loss design of seqAlignATAC against several widely used domain adaptation methods, including MMDDziugaite et al. (2015), CORALSun and Saenko (2016), and CDANLong et al. (2018), on cross-platform cell-type prediction tasks. As shown in Figure 3, seqAlignATAC consistently achieves the highest accuracy and macro F1 across most dataset pairs. These results demonstrate that our graph-regularized and dynamically adversarial alignment strategy provides improved robustness under distributional shift.

Compared with traditional feature-alignment methods such as MMD and CORAL, seqAlignATAC benefits from incorporating cell–cell structural information through the graph, enabling local topology-aware alignment rather than relying solely on feature-level distribution matching. Furthermore, the dynamic adversarial mechanism based on a gradient reversal layer adaptively adjusts the alignment strength during training, effectively mitigating instability in early-stage adversarial optimization.

Relative to CDAN, our method better preserves class-level separability in the unlabeled target domain, leading to improved annotation accuracy and generalization without requiring target labels. Overall, these results validate the strength of the proposed domain alignment strategy within seqAlignATAC.

Comparison of GRL Scheduling Strategies

We further compared different scheduling strategies for the gradient reversal layer (GRL), including constant weights ($\lambda = 0.5$ or 1), linear increase, piecewise increase, and the dynamic strategy adopted in this work. As shown in Figure 4, the Sigmoid-based dynamic schedule achieves the best balance between performance and training stability. This confirms that gradually increasing

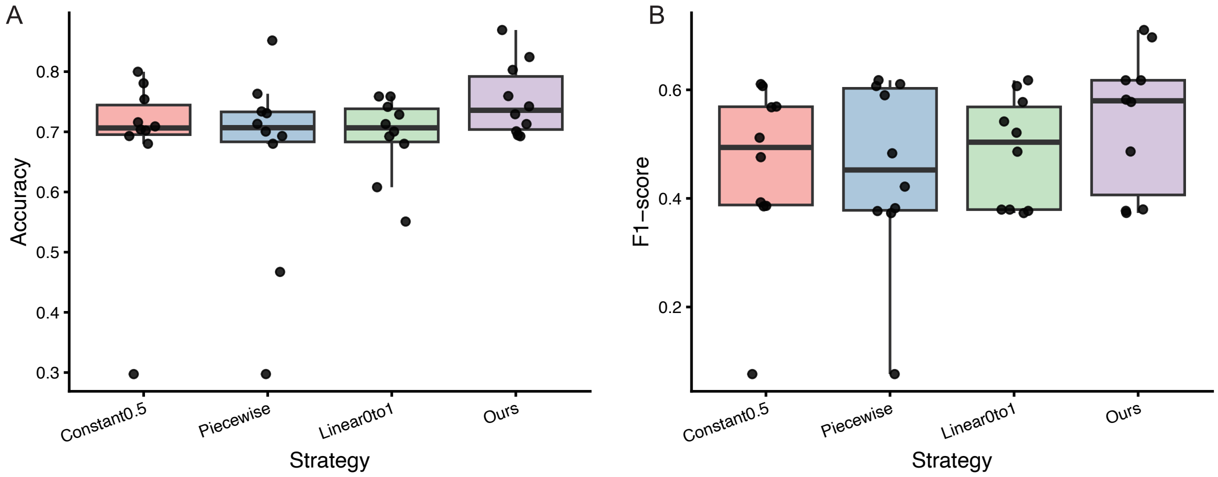

Fig. 4. Comparison of GRL scheduling strategies. (**A**) Accuracy and (**B**) macro F1 across constant scheduling ($\lambda = 0.5$), piecewise increase, linear increase (0→1), and the proposed Sigmoid-based dynamic strategy.

adversarial strength, while keeping feature learning stable in the early stage—leads to more effective domain alignment and overall better performance.

The sensity of λ

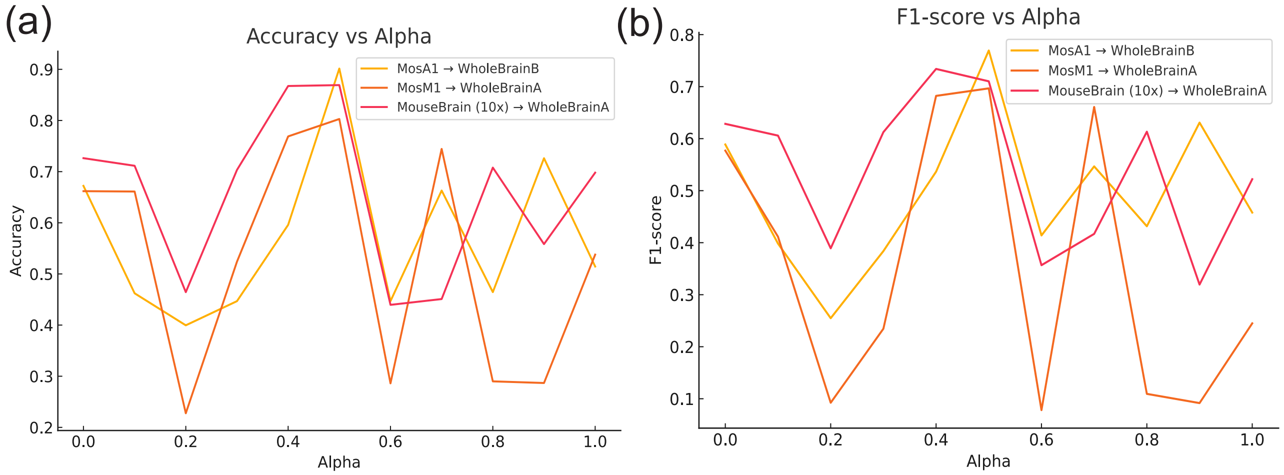

Fig. 5. Ablation analysis on the domain adversarial loss weight α in the total loss. (a) Accuracy and (b) Macro F1-score on three representative cross-dataset tasks.

To investigate the impact of the hyperparameter λ in seqAlignATAC, which balances the domain-adversarial loss ($\mathcal{L}_{\text{dom}}$) and the classification loss ($\mathcal{L}_{\text{cls}}$), we performed an ablation study evaluating performance across different values of λ (Figure. 5). Specifically, we analyzed the model performance in terms of accuracy and F1-score over three transfer learning scenarios: MosA1 → WholeBrainB, MosM1 → WholeBrainA, and MouseBrain (10x) → WholeBrainA.

Experimental results show that low values of λ (e.g., ≤ 0.3) lead to weak domain alignment, while high values (e.g., ≥ 0.7) hurt classification by overemphasizing invariance. Moderate values ($0.4 \sim 0.5$) achieve the best trade-off, with $\lambda = 0.5$ performing best in MosA1→WholeBrainB and MouseBrain(10x)→WholeBrainA. These findings highlight the need for task-specific tuning of λ.

In summary, these findings demonstrate the necessity of carefully selecting the domain-adversarial weight parameter λ to achieve effective domain adaptation and accurate cell type annotation.

Evaluating the Impact of Domain Adaptation in SeqAlignATAC

To assess the importance of the graph domain adaptation component in the SeqAlignATAC framework, we conducted an ablation study comparing two variants: (i) **Embedding(NT)+GDA**, which uses the neural representation extracted in Step 1 followed by the DA-based classification module, and (ii) **Embedding(NT)+KNN**, which replaces the domain adaptation module with a

Table 6. Performance comparison (Accuracy and Macro-F1) across multiple tasks

Task (Metric)	Embedding(NT)+GDA	Embedding(NT)+KNN
MosA1 → WholeBrainA (Acc)	0.701	0.647
MosA1 → WholeBrainA (Macro-F1)	0.578	0.552
WholeBrainA → MosA1 (Acc)	0.824	0.705
WholeBrainA → MosA1 (Macro-F1)	0.582	0.281
MosA1 → WholeBrainB (Acc)	0.902	0.610
MosA1 → WholeBrainB (Macro-F1)	0.769	0.539
WholeBrainB → MosA1 (Acc)	0.804	0.722
WholeBrainB → MosA1 (Macro-F1)	0.455	0.320
MosM1 → WholeBrainA (Acc)	0.803	0.655
MosM1 → WholeBrainA (Macro-F1)	0.697	0.561
WholeBrainA → MosM1 (Acc)	0.759	0.662
WholeBrainA → MosM1 (Macro-F1)	0.380	0.276
MosP1 → WholeBrainA (Acc)	0.713	0.675
MosP1 → WholeBrainA (Macro-F1)	0.618	0.577
WholeBrainA → MosP1 (Acc)	0.729	0.617
WholeBrainA → MosP1 (Macro-F1)	0.373	0.270
MouseBrain(10x) → WholeBrainA (Acc)	0.8692	0.6682
MouseBrain(10x) → WholeBrainA (Macro-F1)	0.7102	0.5925
WholeBrainA → MouseBrain(10x) (Acc)	0.7423	0.7033
WholeBrainA → MouseBrain(10x) (Macro-F1)	0.4864	0.3262
MouseBrain(10x) → WholeBrainB (Acc)	0.6944	0.6392
MouseBrain(10x) → WholeBrainB (Macro-F1)	0.6176	0.5730
WholeBrainB → MouseBrain(10x) (Acc)	0.6927	0.7008
WholeBrainB → MouseBrain(10x) (Macro-F1)	0.3771	0.4075

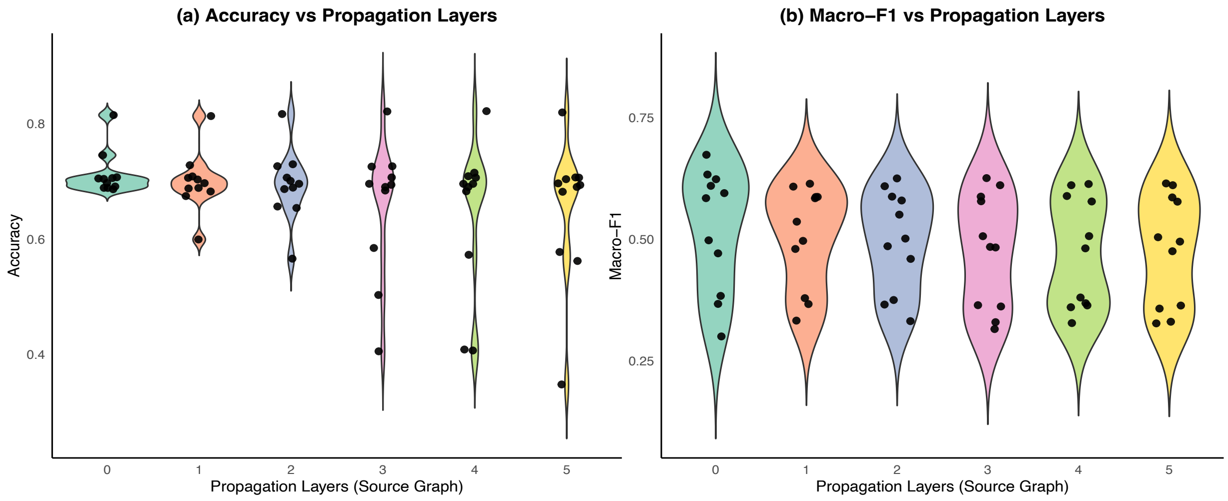

Fig. 6. Effect of varying propagation depth on the **source graph**.

k-nearest neighbor classifier ($k = 6$) operating on the same embeddings. This design enables us to isolate and evaluate the specific contribution of the domain adversarial training in improving cross-domain cell type annotation performance. The evaluation spans 12 transfer tasks involving source and target datasets from different sequencing technologies (e.g., sci-ATAC vs. 10x). As shown in Table 6, Embedding(NT)+DA consistently outperformed the KNN-based variant in both accuracy and macro-F1 across nearly all tasks. Notably, in the MosA1 → WholeBrainB setting, DA achieved 0.9015 accuracy and 0.7694 macro-F1, compared to 0.610 and 0.539 for KNN. Similar improvements are observed in more challenging tasks such as WholeBrainA → MosA1 and MouseBrain(10x) → WholeBrainA, highlighting the domain adaptation module's capability to mitigate representation shift and improve minority cell type recognition. These results demonstrate the necessity of incorporating domain adversarial learning to ensure robust and generalizable cell type annotation across varying biological contexts.

Contribution of the Domain Adaptation Module

To further assess the contributions of the domain adaptation module and the asymmetric propagation strategy, we performed a stepwise ablation study on the second stage of the seqAlignATAC framework. Three configurations were compared: (1) *SourceOnly*, where only source-domain supervision is used; (2) *Adv+Source*, where adversarial loss is added to enable domain adaptation; and (3) *All*, the full seqAlignATAC model with both adversarial alignment and asymmetric propagation.

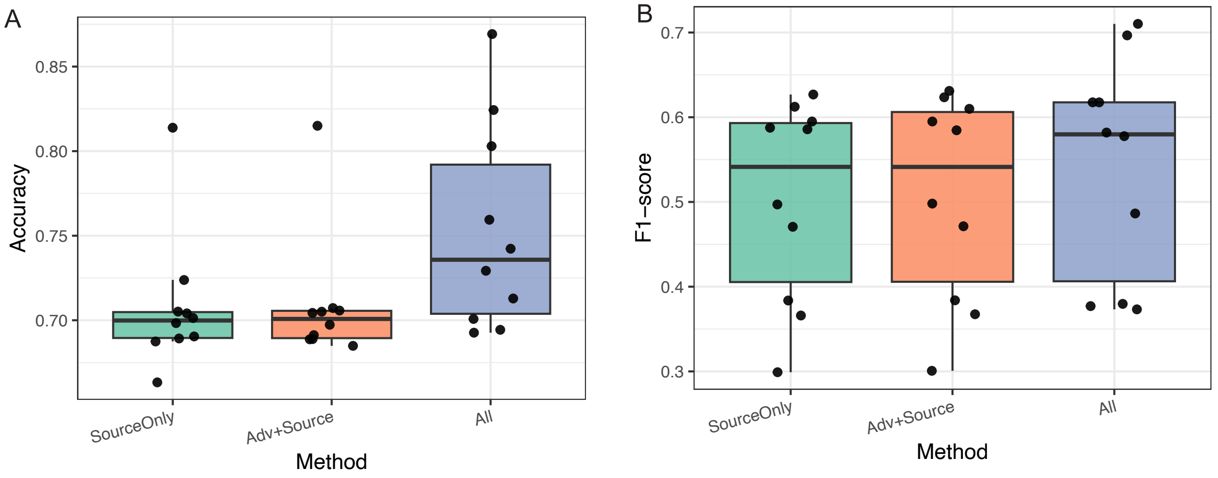

Fig. 7. Ablation study of the domain adaptation module and asymmetric propagation strategy. (**A**) Accuracy and (**B**) macro F1 for three variants: *SourceOnly*, *Adv+Source*, and *All* (full seqAlignATAC).

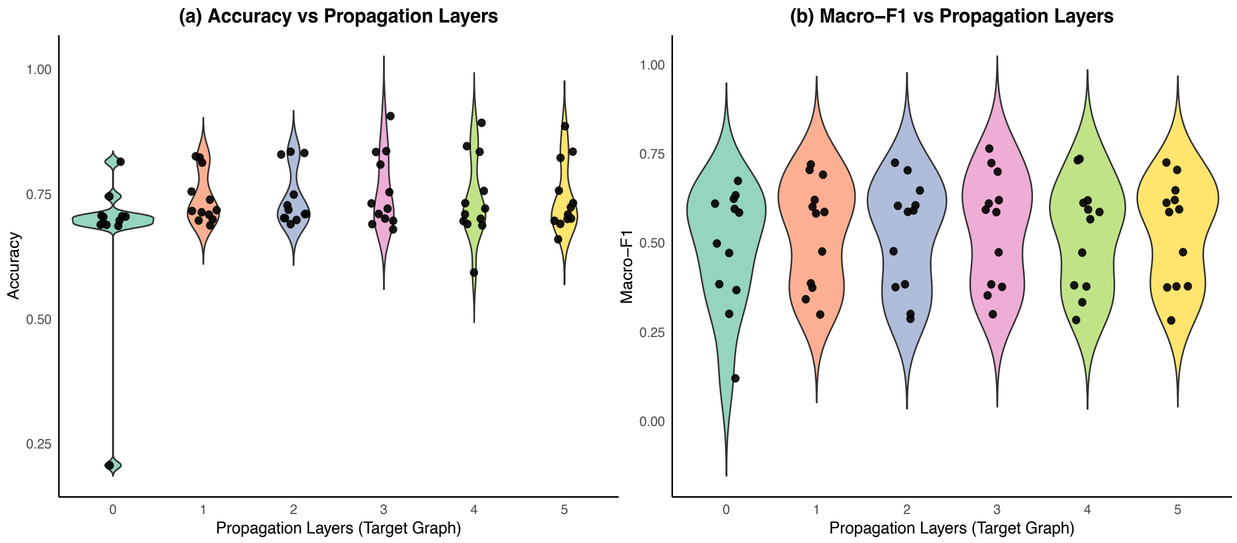

Fig. 8. Effect of varying propagation depth on the target graph.

As shown in Figure 7, adding adversarial loss already improves cross-domain recognition performance, demonstrating the benefit of explicit domain alignment. When the asymmetric propagation strategy is further incorporated, the performance increases more substantially. These results indicate that asymmetric propagation facilitates more stable and efficient information transfer between source and target domains, thereby yielding the strongest performance among all variants.

Evaluating Asymmetric Propagation Depth for Source and Target Graphs

To validate the rationality of our architectural design—shallow propagation on the source graph and deep propagation on the target graph—we conducted ablation studies to investigate how the number of GCN propagation layers affects the cross-graph annotation performance.

As shown in Figure 6, we fixed the propagation depth of the target graph to 0 and varied the number of propagation layers on the source graph from 0 to 5. Experimental results demonstrate that increasing the number of propagation layers on the source graph does not lead to performance improvement. On the contrary, it often results in a noticeable drop in both accuracy and Macro-F1 scores. This confirms the over-smoothing problem inherent in GCNs, where stacking too many propagation layers causes node features to become overly similar, reducing their discriminative power and thus impairing the model's representation and transferability. In fully supervised settings, shallow propagation is sufficient to extract meaningful features from the source graph, while deeper propagation introduces redundant or noisy information.

In contrast, as shown in Figure 8, we fixed the source graph propagation depth to 0 and varied the propagation depth on the target graph from 0 to 5. Results reveal that increasing the propagation depth on the target graph—especially in the range of $K = 3$ to $K = 4$—leads to consistent performance gains across multiple cross-platform tasks. This suggests that deeper propagation

helps capture richer contextual information in the absence of supervision, thereby enhancing the expressiveness and transferability of node features.

In summary, the proposed asymmetric design—shallow propagation for the source graph and deep propagation for the target graph—is both reasonable and effective. It maintains stable feature representation in the supervised source domain while enhancing contextual feature extraction in the unlabeled target domain, ultimately achieving robust performance for cross-graph cell type annotation.

References

Chen, M., Wei, Z., Huang, Z., Ding, B., and Li, Y. (2020). Simple and deep graph convolutional networks. In *International Conference on Machine Learning*, pages 1725–1735. PMLR.

Cover, T. M. and Hart, P. E. (1967). Nearest neighbor pattern classification. *IEEE Transactions on Information Theory*, 13(1):21–27.

Dziugaite, G. K., Roy, D. M., and Ghahramani, Z. (2015). Training generative neural networks via maximum mean discrepancy optimization. *arXiv preprint arXiv:1505.03906*.

Fernández, A., Garcia, S., Herrera, F., and Chawla, N. V. (2018). Smote for learning from imbalanced data: progress and challenges, marking the 15-year anniversary. *Journal of artificial intelligence research*, 61:863–905.

Hamilton, W., Ying, Z., and Leskovec, J. (2017). Inductive representation learning on large graphs. *Advances in neural information processing systems*, 30.

Jiang, Y., Hu, Z., Lynch, A. W., Jiang, J., Zhu, A., Zeng, Z., Zhang, Y., Wu, G., Xie, Y., Li, R., et al. (2023). scatanno: automated cell type annotation for single-cell atac sequencing data. *bioRxiv*. doi:10.1101/2023.06.01.543296.

Kimmel, J. C. and Kelley, D. R. (2021). Semisupervised adversarial neural networks for single-cell classification. *Genome research*, 31(10):1781–1793.

Lin, Y., Wu, T.-Y., Wan, S., Yang, J. Y., Wong, W. H., and Wang, Y. R. (2022). scjoint integrates atlas-scale single-cell rna-seq and atac-seq data with transfer learning. *Nature biotechnology*, 40(5):703–710.

Long, M., Cao, Z., Wang, J., and Jordan, M. I. (2018). Conditional adversarial domain adaptation. *Advances in neural information processing systems*, 31.

Ma, W., Lu, J., and Wu, H. (2023). Cellcano: supervised cell type identification for single cell atac-seq data. *Nature Communications*, 14(1):1864.

Sun, B. and Saenko, K. (2016). Deep coral: Correlation alignment for deep domain adaptation. In *European Conference on Computer Vision*, pages 443–450. Springer.

Veličković, P., Cucurull, G., Casanova, A., Romero, A., Lio, P., and Bengio, Y. (2017). Graph attention networks. *arXiv preprint arXiv:1710.10903*.

Zeng, Y., Luo, M., Shangguan, N., Shi, P., Feng, J., Xu, J., Chen, K., Lu, Y., Yu, W., and Yang, Y. (2024). Deciphering cell types by integrating scatac-seq data with genome sequences. *Nature Computational Science*, 4(4):285–298.
